# The Shining Star of the Last Decade in Regional Anesthesia Part-I: Interfascial Plane Blocks for Breast, Thoracic, and Orthopedic Surgery

**DOI:** 10.5152/eurasianjmed.2022.22321

**Published:** 2022-12-01

**Authors:** Ali Ahiskalioglu, Ahmet Murat Yayik, Erkan Cem Celik, Muhammed Enes Aydin, Bahadir Ciftci, Elif Oral Ahiskalioglu, Bora Bilal, Madan Narayanan, Serkan Tulgar

**Affiliations:** 1 Department of Anaesthesiology and Reanimation, Atatürk University Faculty of Medicine, Erzurum, Turkey; 2 Clinical Research, Development and Design Application and Research Center, Atatürk University Faculty of Medicine, Erzurum, Turkey; 3 Department of Anaesthesiology and Reanimation, Medipol University Faculty of Medicine, İstanbul, Turkey; 4 Department of Anesthesia and Reanimation, Kahramanmaras Sütçü İmam University Faculty of Medicine, Kahramanmaraş, Turkey; 5 Department of Anaesthesia, Frimley Health NHS Foundation Trust, Frimley, UK; 6 Department of Anesthesiology and Reanimation, Samsun University Faculty of Medicine, Samsun, Turkey

**Keywords:** Interfascial plane block, acute pain, multimodal analgesia, postoperative analgesia

## Abstract

Regional anesthesia has benefits beyond just treating acute postoperative pain. Interfascial plane blocks, which have been very popular with ultrasound in recent years, function primarily by administering a high volume of a local anesthetic to the fascial plane. Contrary to traditional peripheral nerve blocks, the targeted nerve or structure in interfacial plane blocks is not fully defined, and the indications have not been fully revealed yet. Anatomical, cadaveric, and radiological studies show how effective the interfascial plane blocks play a role. This review focuses on the benefits, techniques, indications, and complications of interfascial plane blocks in the context of breast, thoracic, and orthopedic surgery.

**Main Points**

- Regional anesthesia is a crucial component of multimodal analgesia in relieving acute postoperative pain.
- Interfascial plane blocks have become very popular in the last decade with the use of ultrasonography in regional anesthesia practice.
- In cases where neuraxial analgesia cannot be applied or is contraindicated, interfascial plane blocks may be an alternative technique for appropriate surgical procedures.
- Interfascial plane blocks show similar efficacy to neuraxial analgesia, which is the gold standard in postoperative analgesia.

## Introduction

Regional anesthesia is a true component of anesthesia, and they are inseparable. Especially in the last 2 or 3 decades, the practice of regional anesthesia regimens has changed considerably. While there were neuraxial techniques at the beginning,^1^ later on, there was application of extremity blocks with a stimulator.^2^ In the following times, facial blocks with pop-ups^3^ showed up in the practice of regional anesthesia. However, now anesthesiologists go far beyond just performing extremity and neuraxial blocks. Today, by using ultrasound, we can see not only the nerves but also the facial planes where the nerves are located, and USG-guided facial plane blocks have become very popular applications.^4^

When we look at the facial plane blocks that start with Transversus Abdominis Plane (TAP) and become popular with erector spinae plane (ESP), we see many blocks defined differently for each plane and various nomenclatures.^5-7^ The answer to the question of whether facial planes used to deliver local anesthetics (LAs) to target nerves can provide anesthesia/analgesia or provide some perks beyond anesthesia/analgesia is still unknown. Contrary to traditional peripheral nerve blocks, the targeted nerve or structure within interfacial plane blocks is not fully defined, and the indications have not been revealed yet. After this point, the question of “What changes future will bring” appears. It would not be difficult to predict what awaits us and what the future of regional anesthesia will hold in this era when the concepts of artificial intelligence, machine learning, and deep learning have roles in our lives now.^8^

In this review, we will not proceed through the names of the blocks, and we will list the types of regional anesthesia that can be used for different types of surgeries.

## Breast and Thoracic Surgery

Innervation of the breast and the axilla is provided by nerves originating from different regions.^9^ The lateral and median pectoral nerves originating from the brachial plexus provide most of the motor and proprioceptive innervation of the pectoralis major and minor muscles. In the axillary area, intercostobrachial nerves and thoracodorsal and thoracic longus nerves are involved. The second leading nerve group that provides sensory innervation of the breast tissue are the anterior and lateral divisions of the T2-T6 intercostal nerves. The intercostal nerves are a continuation of the ventral rami of the spinal nerve roots and extend from the intervertebral foramen to the sternum. It gives off the lateral cutaneous branch at the midaxillary line which perforates through the intercostal and serratus muscles and divides into posterior and anterior branches which supply the posterolateral and anterolateral chest wall, respectively. The terminal anterior cutaneous nerves provide the sensation of the sternal, parasternal region, and medial half of the breast. The innervation is made complex by multiple anastomosis between adjacent spinal/intercostal nerves. The midline has cross innervation from the contralateral anterior cutaneous divisions of corresponding intercostal nerves. The supraclavicular nerves arising from the superficial cervical plexus provide cutaneous innervation to the superior aspect of the breast.^10^ While blockade of intercostal nerves is essential for thoracic surgery, other branches coming from the brachial plexus are also required to be blocked in breast surgery for effective postoperative analgesia.

Epidural anesthesia and thoracic paravertebral block (TPVB) are the gold standard analgesic methods for breast and thoracic surgery; nevertheless, ultrasound-guided (USG) fascial plane blocks have also become popular in recent years. Intertransverse process block, ESP block (ESPB), retrolaminar block (RLB) applied from the paraspinal region, rhomboid intercostal sub-serratus plane (RISS) block applied from the parascapular region, superficial and deep serratus anterior plane (SAP) blocks (SAPBs), interpectoral plane block, and pectoserratus plane (PSP) block applied from the chest wall area are some of them. In addition, the superficial and deep parasternal intercostal plane (PIP) blocks are used for sternotomy and sternal surgery and also as a complementary block in patients undergoing breast surgery.

### Retrolaminar Block

**Figure 1. A, B.**
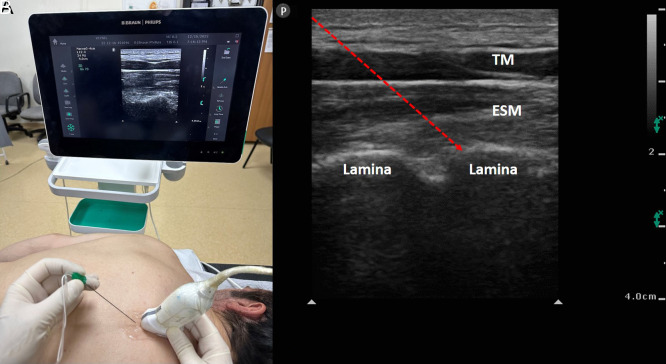
(A) Patient and ultrasound probe position for retrolaminar block procedure. (B) Sonographic anatomy of the block. ESM, erector spinae muscle; TM, trapezius muscle; red arrow, needle.

The retrolaminar block was first described in 2006 as a simple alternative to TPVB.^11^ The aim of the RLB is to only touch the bony vertebral lamina, rather than puncturing the superior costotransverse ligament and entering the paravertebral space. In the USG RLB, local anesthetic is injected into the fascial plane between the posterior surface of the thoracic lamina and the paraspinal muscles (Figure 1). In cadaveric studies, it has been shown that the dye solution spreads anteriorly and spreads to the paravertebral and epidural areas.^12^ It has been shown in clinical studies to provide effective postoperative analgesia in breast surgery and thoracic surgery.^13,14^ In a study conducted on patients who underwent modified radical mastectomy, comparing continuous RLB with paravertebral block (PVB), continuous RLB had a similar effect to PVB except for the first 24 hours.^15^ Furthermore, in a clinical study comparing ESPB and RLB performed with 20 mL of 0.375% levobupivacaine for breast surgery, ESPB was found to be equivalent to and not superior to RLB for postoperative analgesia.^16^ For thoracic surgery, there are 2 different studies comparing RLB with PVB and epidural block. In a retrospective study evaluating 192 minor video-assisted thoracic surgery (VATS) patients, RLB were non-inferior to epidural block.^14^ However, in another study, PVB and RLB were compared in VATS, postoperative pain scores and opioid consumption were lower in patients who underwent PVB.^17^

### Erector Spinae Plane Block

**Figure 2. A, B.**
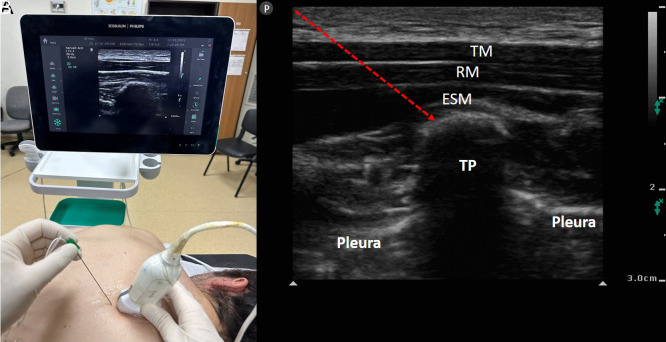
(A) Patient and ultrasound probe position for thoracic erector spinae plane block procedure. (B) Sonographic anatomy of the block. ESM, erector spinae muscle; RM, rhomboid muscle; TM, trapezius muscle; TP, transverse process; red arrow, needle.

Ultrasound-guided ESPB was first reported by Forero et al^18^ and was described for chronic thoracic neuropathic pain in 2016. Erector spinae plane block has since been widely used both in the management of acute postoperative pain and chronic pain.^5,6,19-31^ The ESPB can be applied in a wide area from the cervical to the sacral region along the erector spinae muscle (ESM). It is typically performed by administering local anesthetic between the tips of the vertebral transverse processes and the ESM (Figure 2). The local anesthetic spreads in the cranio-caudal direction to the 3-6 vertebral levels, but the medial-to-lateral spread is limited to the attachments of the ESM to the ribs and thoracolumbar fascia. Though the mechanism of spread into the paravertebral space from an ESPB is unclear, it is postulated from cadaveric studies using high-resolution computed tomography (CT), the spread is through the inter-transverse connective tissue along the dorsal rami and accompanying blood vessels. The local anesthetic spreads anteriorly from the injected plane, passes into the paravertebral area and affects the dorsal and ventral rami of the spinal nerves, and hence provides sensory and sympathetic blockade.^32^ There are many studies and subsequent meta-analyses showing the effectiveness of ESPB in breast surgery and thoracic surgery. Thirty-two articles including 6 randomized controlled studies in a review evaluating ESPB compared with tumescent anesthesia or no block in breast surgery found ESPB can decrease postoperative pain and opioid consumption.^33^ In another meta-analysis of 14 randomized controlled studies comparing ESPB and without a block for postoperative analgesia in breast and thoracic surgery patients, it was shown that ESPB not only significantly improved pain scores up to 24 hours at postoperative time points but also reduced 24-hour opioid consumption. Furthermore, when ESPB was compared with PVB, the analgesic efficacy of ESPB was similar to PVB in pain scores, 24-hour opioid consumption, and postoperative nausea and vomiting (PONV)rates.^34^ Erector spinae plane block is also used in the treatment of chronic pain in the thoracic region with recurrent blocks or catheter applications. It is effective in cancer pain involving a single hemithorax or postherpetic neuralgia.^35,36^

### Rhomboid Intercostal Block

**Figure 3. A-D.**
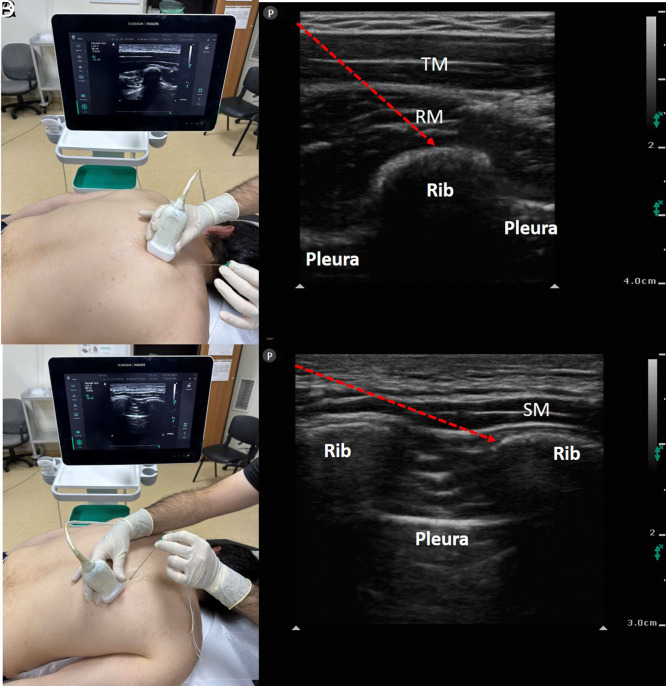
(A–C) Patient and ultrasound probe position for rhomboid intercostal sub-serratus block procedure. (B–D) Sonographic anatomy of the block. RM, rhomboid muscle; SM, serratus muscle; TM, trapezius muscle; red arrow, needle.

The rhomboid intercostal block (RIB) is performed in the area known as the auscultation triangle in the posterior chest wall region. The RIB is performed with the injection of local anesthetic in the plane between the rhomboid and the intercostal muscles. The RIB provides anesthesia between T2 and T7 dermatomes (Figure 3). The recent modification of this block, which is named the RISS block, affects T2-T11 dermatomes. Considering the dermatomal areas where it is effective, this block can be used in breast and axillary surgery, thoracic surgery, and upper abdominal surgery. There are publications showing the effectiveness of RIB in modified radical mastectomy, breast reduction surgery, and in the treatment of chronic pain after breast surgery.^37-39^ For the thoracic region, its effectiveness has been demonstrated in VATS and multiple rib fractures in the lateral region.^40-43^ A meta-analysis of studies that evaluated RIB and no block in patients undergoing breast and thoracic surgery showed that patients who underwent RIB had lower pain scores, opioid consumption, and PONV rates.^44^ In another study comparing the efficacy of RISS, ESP, and SAPB in patients undergoing VATS, it was identified that the patients who underwent RIB and ESPBs had lower opioid consumption and dynamic pain scores in the first 24 hours postoperatively compared to SAPB.^45^

### Serratus Anterior Plane Block

**Figure 4. A, B.**
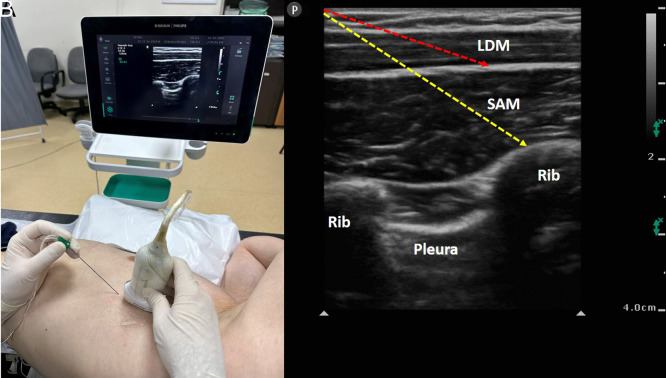
(A) Patient and ultrasound probe position for serratus anterior plane block procedure. (B) Sonographic anatomy of the block. LDM, latissimus dorsi muscle; SAM: serratus anterior muscle; red arrow, needle direction for superficial serratus anterior plane block; yellow arrow, needle direction for deep serratus anterior plane block.

This block is performed by injecting LA between the serratus anterior muscle and the rib (deep SAPB) or between the serratus anterior muscle and the latissimus dorsi muscle (superficial SAPB) in the area where the fourth ribs intersect with the midaxillary line (Figure 4). In the SAPB, T2-T8 intercostal nerves and long thoracic and thoracodorsal nerves are blocked. The SAPB has mainly been shown to be effective in oncological breast surgery, breast reduction surgery, thoracic surgery, anterolateral rib fractures, and chest wall chronic pain treatment.^46-49^ Blanco et al.^50^ in a small study on female volunteers, compared superficial and deep SAPB. Each volunteer was given 2 injections with a deep SAPB on one side and a superficial SAPB on the other, with LA and gadolinium. In the dermatological examination and magnetic resonance imaging performed 30 minutes after the injection, it was observed that a large area of thoracic dermatomes was anesthetized in all patients. In their study, a larger area and a longer duration of sensory loss were reported in the superficial SAP compared to the deep SAP. There are many randomized controlled studies showing the efficacy of SAPB in breast and thoracic surgery. In a meta-analysis of studies evaluating the efficacy of SAP in breast cancer surgery, it was shown that SAPB reduces intraoperative fentanyl consumption, reduces postoperative analgesic requirements, and reduces postoperative nausea and vomiting. When compared to TPVB, SAPB was not worse for the all evaluated parameters.^48^ Similarly, in a meta-analysis evaluating the efficacy of SAPB in thoracic surgery, SAPB reduced both pain scores and postoperative 24-hour opioid consumption, in addition, there was a lower incidence of PONV in the SAPB group.^51^

### Interpectoral Plane Block and Pectoserratus Plane Block

**Figure 5. A, B.**
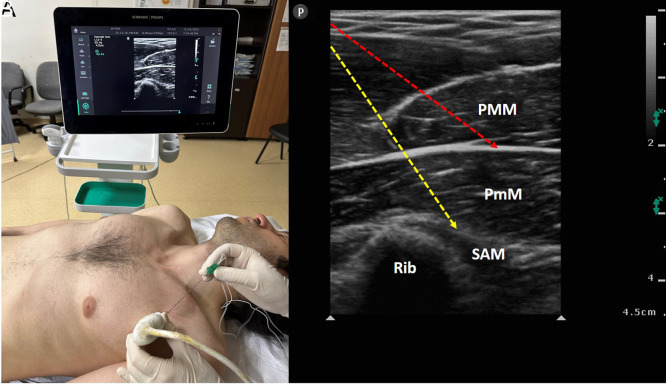
(A) Patient and ultrasound probe position for interpectoral plane block and pectoserratus plane block procedure. (B) Sonographic anatomy of the block. PMM, pectoralis major muscle; PmM, pectoralis minor muscle; SAM, serratus anterior muscle; red arrow, needle direction for interpectoral plane block; yellow arrow, needle direction for pectoserratus plane block procedure.

Interpectoral plane block (previously known as PECS-I block) is applied by injecting LA between the pectoralis major and pectoralis minor muscles (Figure 5). This injection point is the fascial plane where the pectoral branch of the thoraco-acromial artery and the lateral pectoral nerve are located. The medial pectoral nerve is located in the inferolateral region of this injection point. The pectoral nerves have no cutaneous innervation, they only innervate the pectoral muscles. But it is still used to manage pain caused by stretching or injury of the pectoral muscles.^52^ Pectoserratus plane block (formerly called PECS-II block) is performed by injecting LA between the pectoralis minor and serratus anterior muscles at the intersection of the third or fourth rib in the anterior axillary line. The dermatomal area affected by the PSP block is similar to the SAPB and it is covered the intercostobrachial nerve, long thoracic nerve, and third-sixth intercostal nerves.^53^ Interpectoral plane and PSP blocks were first described as an alternative to paravertebral block, providing effective analgesia in breast surgery. And also it was widely used in thoracic surgery, cardiac surgery, and post-surgical chronic pain management. In a meta-analysis that included 1026 patients and 16 studies, in patients undergoing mastectomy and other breast surgery, comparing the effects of PSP block and no block, the meta-analysis provided moderate-to-high evidence that PSP blocks provide postoperative analgesia after breast surgery.^54^ In another meta-analysis comparing PSP block with control, LA infiltration, ESPB, and paravertebral block in breast cancer surgery, PSP block reduces intraoperative and postoperative opioid consumption compared to control and ESPB, and PSP provides better pain relief compared to paravertebral block.^55^ Furthermore, PSP blocks not only reduce the effect on acute postoperative pain but also reduce the incidence of chronic pain after breast surgery.^56^ For thoracic surgery, it has been shown to be effective as a part of multimodal analgesia in thoracoscopic surgery and minimally invasive pectus excavatum surgery.^57,58^

### Parasternal Intercostal Plane Block

**Figure 6. A-D.**
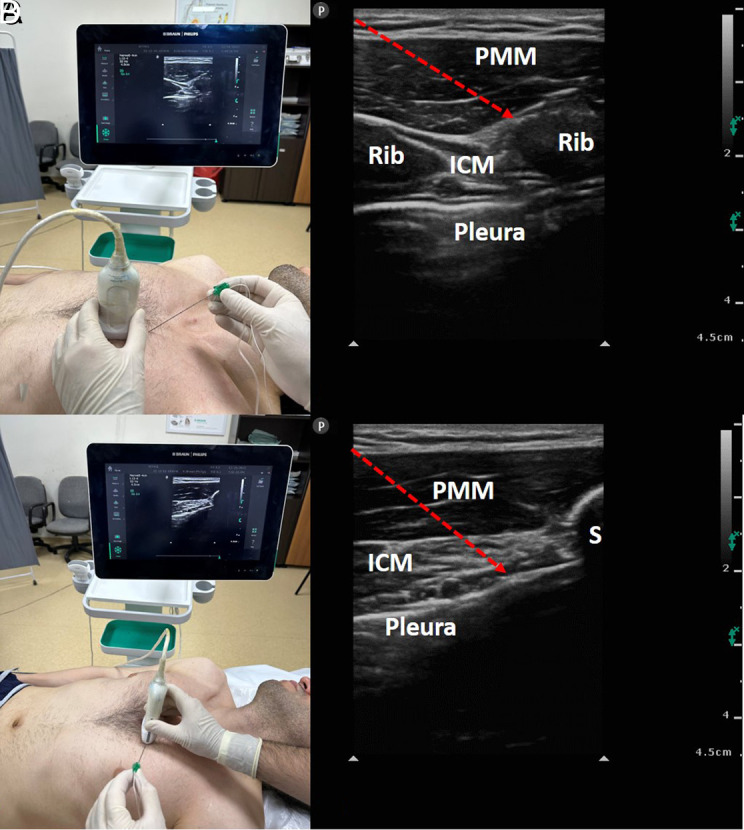
(A) Patient and ultrasound probe position for superficial parasternal intercostal plane block. (B) Sonographic anatomy of the superficial parasternal intercostal plane block. (C) Patient and ultrasound probe position for deep parasternal intercostal plane block. (D) Sonographic anatomy of the deep parasternal intercostal plane block. PMM, pectoralis major muscle; red arrow, needle direction.

Parasternal intercostal plane blocks are performed with local anesthetic injection between the internal intercostal muscles and the pectoralis major muscle (superficial PIP) (Figure 6A and B) or between the internal intercostal muscles and the transverse thoracic muscle (deep PIP) (Figure 6C and D) in the parasternal area at the level of the third and fourth ribs. It blocks the anterior cutaneous branches of the 2-6 intercostal nerves. These blocks are frequently used for postoperative analgesia in sternal surgery and cardiac surgery with sternotomy. Parasternal intercostal plane blocks are also used in combination with other lateral wall blocks (SAPB, pectoserratus block) to provide complete analgesia in breast surgery.^59-62^

## Orthopedic Surgery

Pain management is a critical issue after orthopedic surgery. Patients may suffer moderate-to-severe pain due to bone and muscle operations in orthopedic surgeries.^63^ Regional anesthesia techniques are used for this aim as a part of multimodal analgesia. Techniques such as interscalene nerve block and epidural analgesia (EA) are gold standard methods; however, they have complications such as diaphragmatic paralysis for interscalene block (ISB), and dural puncture, hypotension, headache for EA.^64-66^ In addition, they have some technical difficulties in applications. Therefore, simple and safe methods are needed in daily applications. The rising star “interfascial plane blocks” are very popular in anesthesia practice nowadays. Thanks to the use of ultrasound novel plane blocks have been described recently.^7^ In this section, our aim is to refer to fascial plane blocks that can be used for postoperative analgesia management following orthopedic surgeries.

### Erector Spinae Plane Block

**Figure 7. A, B.**
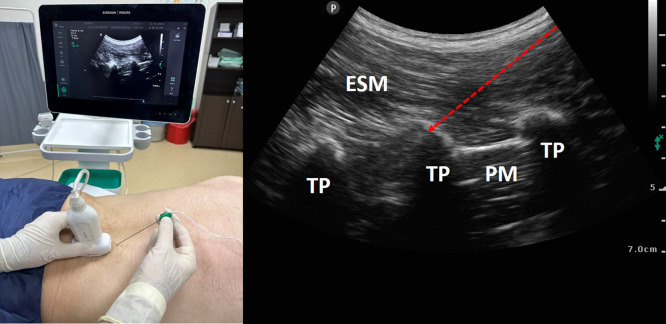
(A) Patient and ultrasound probe position for lumbar erector spinae plane block procedure. (B) Sonographic anatomy of the block. ESM, erector spinae muscle; PM, psoas muscle; TP, transverse process; red arrow, needle.

The LA is performed between the transverse process and ESM into the plane deep into the ESM (Figure 7). The LA runs a long way in the deep fascia of ESM. Erector spinae plane block provides a large sensorial blockade due to this plane contains dorsal and ventral rami.^5,6,25,32,67-69^ In addition, it has been reported that ESPB spreads epidural and paravertebral spaces.^31^ Since ESPB has these features, it was classified as a paraspinal block. High-thoracic/cervical ESPB may be used for pain relief after arthroscopic shoulder surgery, upper extremity surgery, shoulder disarticulation surgery, and for chronic shoulder pain.^70-73^ The sensorial innervation of the shoulder and upper extremity is provided by the C4-C8 segments of the brachial plexus. It has been reported that the T2-level ESPB spreads over the C4-C8 in CT images. The ESPB may be an alternative analgesic technique in patients with pulmonary comorbidities after shoulder surgery due to its phrenic nerve-sparing effect. Ma et al^74^ performed high-thoracic ESPB for proximal humerus surgery and total shoulder arthroplasty surgery, and they reported that ESPB has a motor-sparing effect. Diwan et al^75^ reported ESPB as a phrenic nerve-sparing block for shoulder surgeries. Ekinci et al^71^ performed ESPB for upper extremity surgery and they reported low pain scores and no additional analgesic drug needed. Ciftci et al^70^ performed T2-level ESPB for arthroscopic shoulder surgery in their randomized study, and they reported that ESPB provided better pain relief compared to the sham group. Kapukaya et al^76^ compared the ESPB and ISB after arthroscopic shoulder surgery, and they reported ISB in the first 4 hours postoperatively. The ESPB may be a better choice for patients undergoing shoulder surgery, especially in patients with pulmonary insufficiency and anatomical difficulties. Although many studies show that lumbar ESPB is effective in hip surgery,^69,77^ some studies argued that it is not effective.^78^ For lumbar ESP to be effective in hip surgery, it must be done at L3 and below levels, and the volume of LA must be high.

### Deep Supraspinatus Muscle Plane Block

**Figure 8. A, B.**
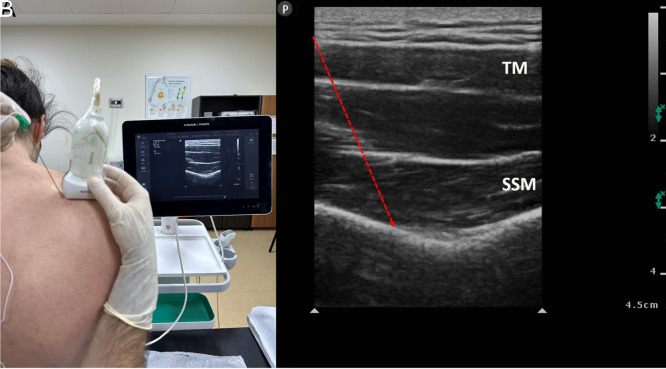
(A) Patient and ultrasound probe position for deep supraspinatus muscle plane block procedure. (B) Sonographic anatomy of the block. SSM, supraspinatus muscle; TM, trapezius muscle; red arrow, needle.

Kose et al^79^ described the deep supraspinatus muscle plane block (DSMPB), which involves the injection of LA into the plane between the supraspinatus muscle and the posterior scapula (Figure 8). They performed the DSMPB on the lateral aspect of the scapula below the muscle. Deep supraspinatus muscle plane block targets the suprascapular nerve branches.^79,80^ The suprascapular nerve innervates the cephalad cutaneous part of the shoulder. Kose et al^79^ performed DSMPB on a patient with chronic shoulder pain. They reported pain relief and a pain-free period of 12 weeks. Kose et al^81^ performed a radiological evaluation of DSMPB, and they reported significant radiocontrast spread within the whole supraspinous fossa and partially spread into the infraspinous fossa. Additionally, dye spread in the supraspinous fossa has been reported in their cadaveric investigation.^82^ Altiparmak et al^83^ performed DSMPB for post-laparoscopic shoulder pain, and they reported effective analgesia in their patient. Öksüz et al^84^ performed DSMPB for rescue analgesia after shoulder surgery, and they reported pain relief after the block. The DSMPB may be performed for acute and chronic shoulder pain.

### Serratus Anterior Plane Block

Serratus anterior plane block was first defined by Blanco et al^50^ in 2013. Blanco et al performed LA between latissimus dorsi and serratus anterior muscles at the level of fourth-fifth ribs for SAPB. Serratus anterior plane block provides analgesia in the hemithorax. Serratus anterior plane block is commonly used for analgesia after thoracic surgeries, pain relief in rib fractures, and breast surgeries.^40,47,49,85^ Serratus anterior plane block may be also used in shoulder surgery with an adequate thoracic sensorial blockade. Demir et al^86^ compared ISB and ISB + SAPB in patients who underwent arthroscopic shoulder surgery. In their randomized study, they performed 30 mL LA in the 2 groups. They reported that SAPB + ISB increased the quality of surgical anesthesia and reduced the need for intraoperative sedo-analgesia. Serratus anterior plane block may be used with ISB for anesthesia management during shoulder surgery.

### Pericapsular Nerve Group Block

**Figure 9. A, B.**
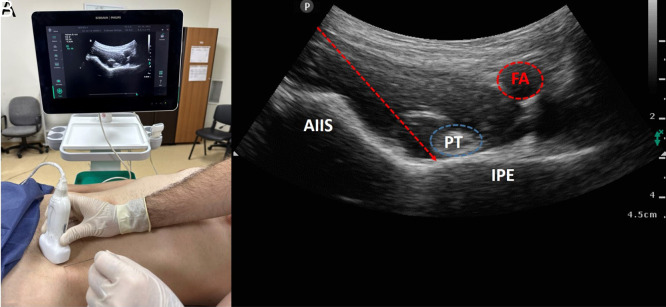
(A) Patient and ultrasound probe position for pericapsular nerve group block procedure. (B) Sonographic anatomy of the block. AIIS, anterior inferior iliac spine; FA, femoral artery; IPE, iliopubic eminence; PT, psoas muscle tendon; red arrow, needle.

Pericapsular nerve group (PENG) block was first described by Girón-Arango et al^87^ in 2018. They performed PENG block for hip fracture in 5 patients. They reported reduced pain scores compared to baseline and no motor weakness of the quadriceps muscle. Pericapsular nerve group is performed into the fascial plane between the psoas tendon and pubic ramus with an in-plane approach by using a convex probe (Figure 9). The anatomical landmarks are the anterior inferior iliac spine, pubic ramus, iliopubic eminence, iliopsoas muscle and tendon, the femoral artery, and pectineus muscle. Pericapsular nerve group block targets the articular branches of the hip capsule. The anterior hip capsule is mainly innervated by the articular branches of the femoral nerve and accessory obturator nerve. Pericapsular nerve group mainly provides analgesia for the anterior part of the hip.^87^ However, in a cadaveric study, it was reported that PENG may spread to the joint surface and posterior of the hip.^88^ It was reported that a high-volume PENG block may act as a lumbar plexus block.^89^ Ahiskalioglu et al^90^ used the PENG for surgical anesthesia of the medial thigh. Ciftci et al^91^ reported that high-volume PENG may result in motor blockade due to the spread of the femoral nerve and obturator nerve. Pericapsular nerve group block may be effectively used for postoperative analgesia management in patients who underwent hip surgery. In addition, by using high volumes, PENG can be an alternative method for postoperative analgesia after knee surgery.

### Suprainguinal Fascia Iliaca Block

**Figure 10. A, B.**
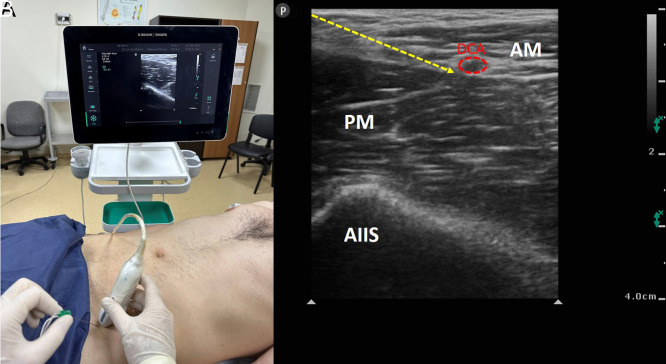
(A) Patient and ultrasound probe position for suprainguinal fascia iliaca block procedure. (B) Sonographic anatomy of the block. AIIS, anterior inferior iliac spine; AM, abdominal muscles; DCA, deep circumflex iliac artery; FA, femoral artery; PM, psoas muscle; yellow arrow, needle.

Fascia iliaca block (FIB) contains the injection of LA in the plane between the fascia iliaca and the iliacus muscle (Figure 10), which includes the femoral nerve and lateral femoral cutaneous nerve (LFCN).^92^ It is used for the anesthesia/analgesia of the hip, knee, and thigh.^93^ Ultrasound-guided FIB has 2 approaches: suprainguinal and infrainguinal approaches. Hebbard et al^93^ reported that LA is injected directly into the iliac fossa during suprainguinal fascia iliaca block (SFIB). The needle is directed under the fascia iliaca from below the inguinal ligament. Thus, the needle tip passes superior to the ligament. It has been shown that injection with suprainguinal technique leads to a wide spread in the iliac fossa of cadavers. The SFIB may allow lower LA volumes to block the femoral nerve and LFCN in patients. In the literature, SFIB has been used for total hip arthroplasty, knee surgery, and pediatric knee/hip surgery.^94-98^ The SFIB and PENG may be used for ease of positioning during spinal anesthesia in patients with hip fractures.^99^
